# Discitis and Osteomyelitis Caused by a Migrated Laparoscopic Adjustable Gastric Banding (LAGB) Catheter

**DOI:** 10.7759/cureus.42815

**Published:** 2023-08-01

**Authors:** Tanner Redlin, Andrew Holmes, Megan Albertson

**Affiliations:** 1 Diagnostic Radiology, University of South Dakota Sanford School of Medicine, Sioux Falls, USA; 2 Neuroradiology, Avera McKennan Hospital and University Center, Sioux Falls, USA

**Keywords:** bariatric, lower back pain (lbp), vertebral discitis, adult osteomyelitis, general surgery complication, neuro radiology, general radiology, laparoscopic adjustable gastric band

## Abstract

Laparoscopic adjustable gastric banding (LAGB) is a popular bariatric surgical procedure used to aid in weight loss. Although significant complications may occur after LAGB, they are rare. LAGB causing discitis and osteomyelitis are incredibly rare, with only one other reported case. In this case report, we describe the case of a middle-aged woman who experienced discitis and osteomyelitis due to a disengaged LAGB catheter, which had eroded through her stomach and a part of her cecum.

Overall, this case highlights the rare but potential complication of LAGB causing discitis and osteomyelitis. Patients with a history of LAGB placement should be monitored for this possibility and further investigation is needed to identify and mitigate risk factors.

## Introduction

Back pain is one of the most common clinical complaints with 39% of Americans experiencing back pain in the last three months [1]. The etiology of back pain is broad and includes trauma, degenerative changes, neoplasm, infection, and other referred causes such as aortic aneurysm. Due to the diverse sources of back pain, it is essential to approach back pain with a broad differential diagnosis and obtain a thorough history.

In this case, our patient had an exceedingly rare cause of lower back pain due to medical device failure after a prior bariatric surgery. We present the second reported case of dislodgement and migration of laparoscopic adjustable gastric banding (LAGB) tubing with resultant spinal osteomyelitis and discitis and concomitant erosion of the stomach and cecum [2,3]. Our case illustrates the importance of considering potential spinal complications in patients with a history of LAGB placement or any other peritoneal catheter placement. This case also substantiates existing medical literature regarding this rare complication in patients contemplating LAGB bariatric surgery.

This article was previously presented as a poster at the 2022 ASSR Annual Meeting in February 2022.

## Case presentation

**Figure 1:**
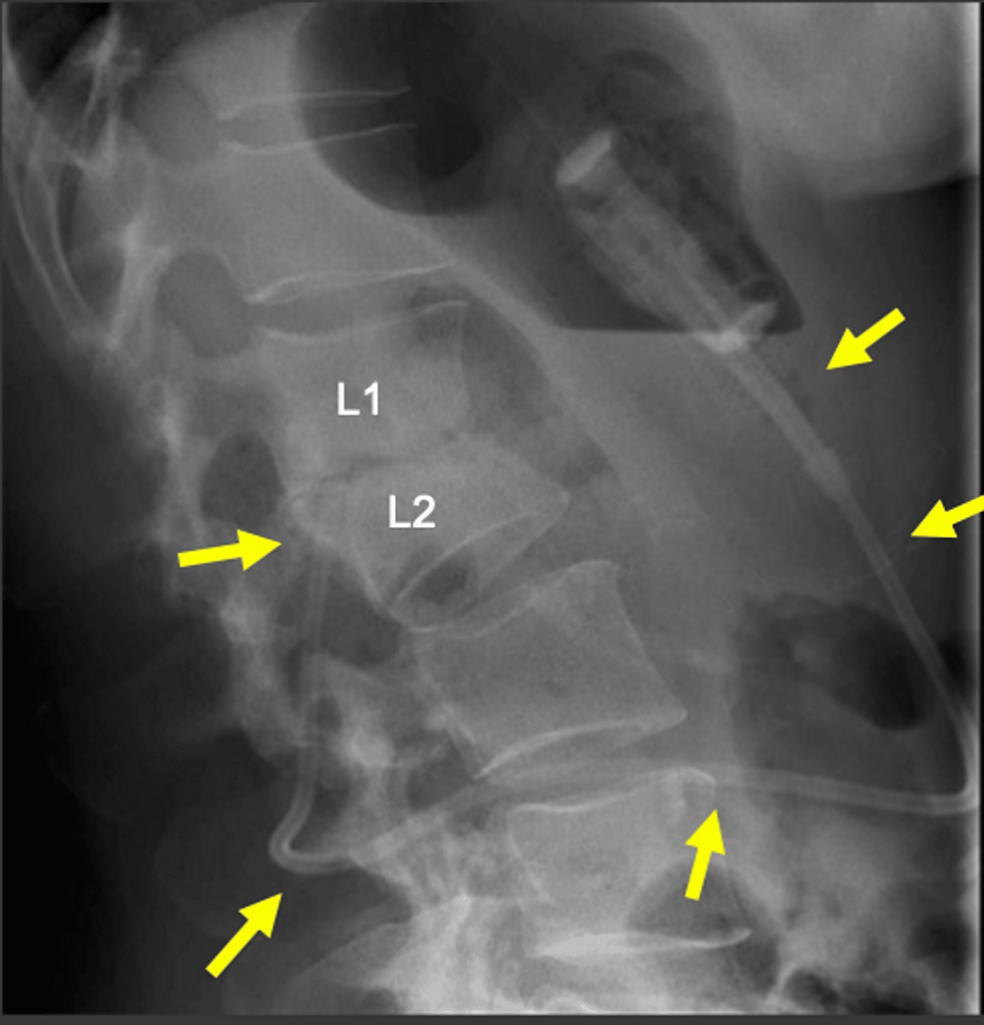
Lumbar spine x-ray showed height loss of the L1-2 intervertebral disc and erosive changes of the adjacent endplates. A catheter fragment is also seen extending from the gastric band to the disc space.

The patient presented to an outpatient clinic with a four-month history of back pain and muscle spasms that were most noticeable when seated or doing twisting motions. She had no radicular symptoms, no gastrointestinal complaints, no recent trauma, and no fever. She was treated conservatively with heat, Tylenol, and physical therapy which provided no relief. Her spasms progressed to the point where she lost control of her bladder, and she was then started on muscle relaxers which helped her symptoms significantly. Further questioning revealed she had a history of fibromyalgia and LAGB placement 13 years prior without any other surgical history. On exam, the patient was mildly tender to palpation over the mid-lumbar spine and the straight raise test resulted in increased pain in the left lower back without radiation. There was mild discomfort noted with the range of motion of both hips, but no weakness was appreciated in the lower extremities. A lumbar spine x-ray was ordered and revealed compression fractures of L1 and L2 as seen in Figure 1.

**Figure 2:**
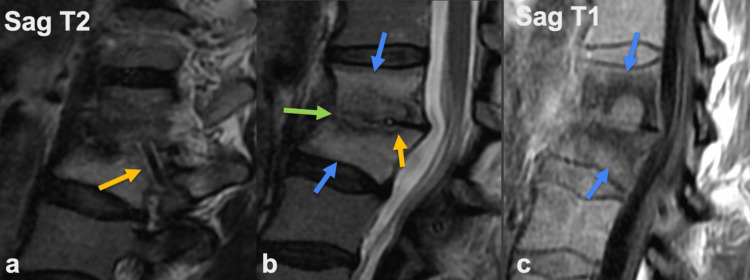
Sagittal T2WI (a, b) and T1WI (c) of the lumbar spine. Catheter fragment (gold arrow) terminating in the L1-2 disc space. Extensive marrow edema (blue arrow) of the L1 and L2 vertebral bodies and destruction of the disc space (green arrow) is consistent with discitis and osteomyelitis.

Subsequent MRI of the lumbar spine showed discitis and osteomyelitis of L1-2 with a catheter fragment terminating in the left disc space as seen in Figures 2a-2c.

**Figure 3:**
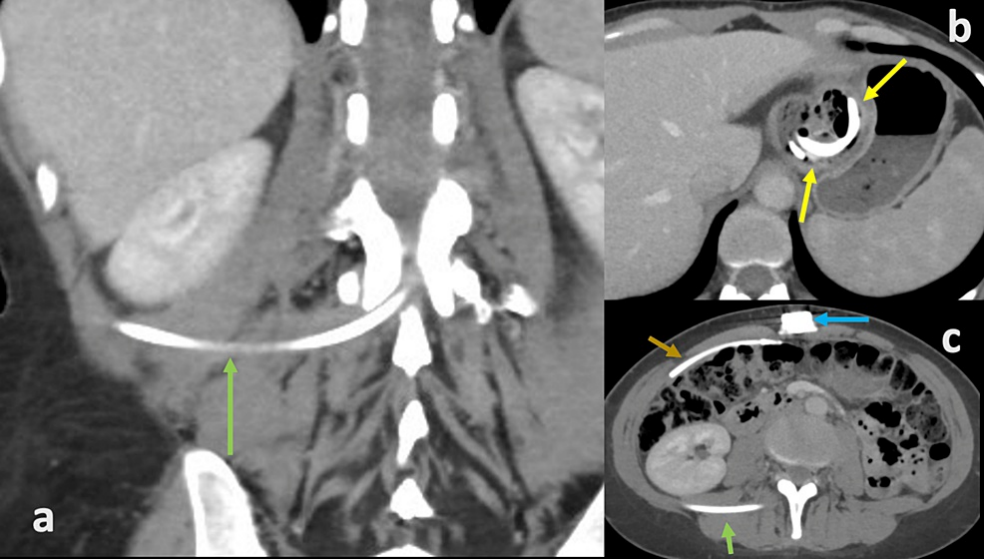
Abdomen/pelvis CT with contrast. Axial (a) and coronal (c) CT images show the catheter fractured (orange arrow) approximately 6 inches from the access port (blue arrow). The catheter segment attached to the band (green arrows) courses through the peritoneal cavity into the right lateral body wall and into the L1-2 disc space. Axial imaging (b) reveals erosion of the gastric band through the stomach wall and into the stomach lumen (yellow arrows).

**Figure 4:**
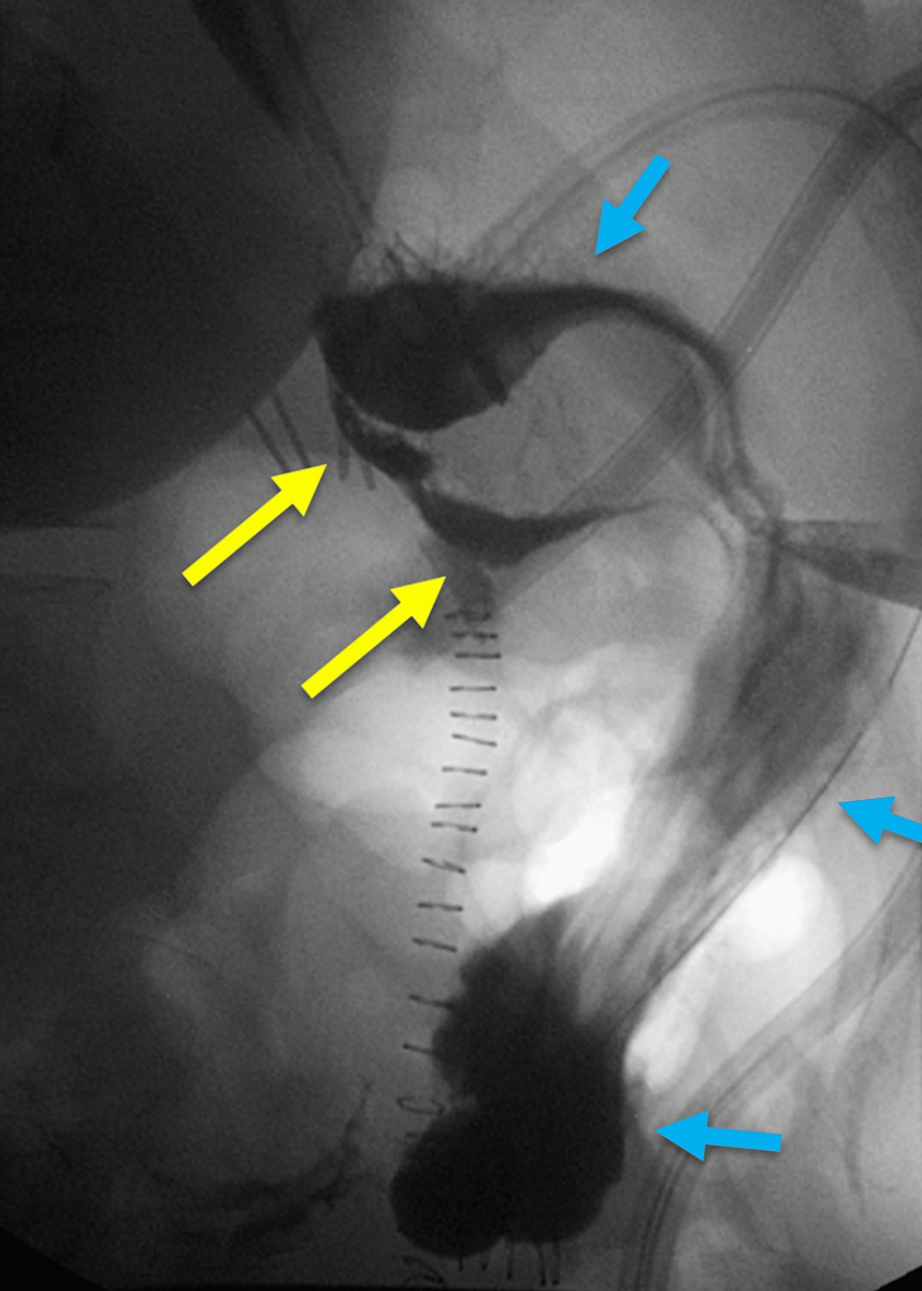
Water-soluble upper gastrointestinal series Fluoroscopic evaluation of the stomach and lower esophagus showed extraluminal oral contrast at the level of the gastric band (yellow arrows) without clear communication into the peritoneal cavity. Contrast is also noted in the stomach lumen (blue arrows).

She had no fevers, chills, or generalized aches. In retrospect, the lumbar spine x-ray did exhibit the broken LAGB catheter terminating in the lumbar spine. CT of the abdomen/pelvis (Figures 3a-3c) and upper GI series (Figure 4) also demonstrated that the LAGB catheter had eroded through the stomach and part of the cecum.

The patient was admitted to a local hospital and infectious disease specialists (ID) were consulted. Initial white blood cell count (WBC) was 8.5 K/µL (4.5-11.0 K/µL), erythrocyte sedimentation rate (ESR) was 51 mm/hr (0-20 mm/hr), and C-reactive protein (CRP) was 0.8 mg/dL (<0.5 mg/dL). Treatment was initiated with IV vancomycin and cefepime and surgical removal of the gastric band port, broken catheter, and repair of the perforated stomach and cecum.

Cultures from a CT-guided disc aspiration/bone biopsy grew Streptococcus constellatus so antibiotics were transitioned to Ertapenem 1g IV daily plus Daptomycin 500 mg IV daily for a total of six weeks. She was discharged home with wound care, acetaminophen for pain, home health skilled nursing, and follow-up appointments with surgery and primary care.

A follow-up MRI of the lumbar spine a few months later showed signs of resolving osteomyelitis/discitis with expected maturation of destructive endplate changes at L1-2, anterior wedging of the L2 vertebral body, kyphosis at the L1-2 level, and significantly decreased adjacent inflammation. There was no evidence of new or recurrent osteomyelitis/discitis or soft tissue abscess.

## Discussion

The prevalence of obesity within the United States has reached alarming rates, with an increase from 30.5% to 42.4% from 2000 to 2018 [4]. Obesity is associated with numerous health complications, including cardiovascular disease, stroke, type 2 diabetes, non-alcoholic fatty liver disease, and osteoarthritis [5]. LAGB is a bariatric surgical option that can aid in weight loss and manage the secondary effects of obesity. However, the use of LAGB has decreased substantially relative to other bariatric surgical options such as the gastric sleeve and Roux-en-Y gastric bypass, according to the American Society of Metabolic and Bariatric Surgery [6]. In 2011, approximately 35% of bariatric surgeries were LAGB procedures and by 2015 the number decreased to 6% [6].

While the incidence of complications during and after LAGB surgery is low, they can occur [2,3]. Minor complications, including pouch dilation (12%), persistent gastroesophageal reflux disease (7%), port prominence (2.5%-6%), and port malfunction (<1%), have relatively low prevalence [4,7]. Major complications are even less frequent and include band slippage (<5%), late port infection (<1%), band erosion (<1%), and stomal obstruction (frequency unknown) [4,7]. Disengagement of LABG tubing accounts for 17%-21% of all complications, but it typically results in abdominal pathology such as stomach erosion [4,7]. In 2020, Iowa reported the first case of LAGB-associated discitis and osteomyelitis [2,3]. Our case shares several similarities with the reported case in Iowa, including a positive straight leg raise test, intact sensation and strength, and maintaining a full range of motion on the initial exam.

## Conclusions

In conclusion, diagnostic imaging is warranted in patients with lumbar pain and muscle spasms who do not respond to conservative treatment. Although spinal complications from intra-peritoneal devices are rare, it is a reminder that iatrogenic causes for infection should always be considered and a thorough history is needed in all patients. Our case illustrates the importance of considering potential spinal complications in patients with a history of LAGB placement and pursuing advanced imaging in patients with atypical pain or lack of short-term improvement. This case also substantiates existing medical literature regarding this extremely rare complication in patients contemplating LAGB bariatric surgery.
